# Early Maternal Serum β-human Chorionic Gonadotropin Measurements After ICSI in the Prediction of Long-term Pregnancy Outcomes: A Retrospective Cohort Analysis

**DOI:** 10.4021/jocmr477e

**Published:** 2011-02-12

**Authors:** Mamdoh A. Eskandar, Mesfer Al-Shahrani, Ayman Shaamash, Mohamed El-Emain, Mutaz Al-Ahmad, Beverly Payodon

**Affiliations:** a Department of Obstetrics and Gynecology and Reproductive Medicine, College of Medicine, King Khalid University, Abha, Saudi Arabia; b Department of Obstetrics and Gynecology and Reproductive Medicine, Saudi Center for Assisted Reproduction, Abha, Saudi Arabia; c Saudi Center for Assisted Reproduction, Abha, Saudi Arabia

## Abstract

**Background:**

Initial low maternal serum β-human chorionic gonadotropin (β-hCG) is a good predictor of early pregnancy demise. Our objective was to determine its predictive value in determining the long-term outcome in ICSI pregnancies.

**Methods:**

A retrospective cohort study was designed at the Saudi Center for Assisted Reproduction. Two hundred and sixty-one women with ICSI pregnancies were followed up from initial β-hCG level determination till the end of pregnancy. Accuracy of early β-hCG in predicting the occurrence of a live-birth, ongoing pregnancy, late miscarriage, ectopic pregnancy and early miscarriage following ICSI was measured.

**Results:**

β-hCG levels were significantly different in pregnancies that reached the stage of an ongoing pregnancy and live-birth as compared to early pregnancy loss. The ROC curves demonstrated a high sensitivity for identifying patients with ectopic pregnancies and early miscarriage (100% and 93.33% respectively). The remaining results ranged from a sensitivity of 69% to 79% and specificity of 62% to 75%.

**Conclusions:**

In ICSI pregnancies, a single early β-hCG may help to identify pregnancies that will reach full-term and delivery.

**Keywords:**

ICSI; Human chorionic gonadotropin; Outcome; Pregnancy

## Introduction

Human chorionic gonadotropin (hCG), being produced and secreted by syncytial trophoblast, is a vital part of early pregnancy. Through the stimulation of luteinizing hormone (LH) receptors, it maintains the corpus luteum and progesterone production. Additionally, since it is produced by the early fetus, and can be measured in maternal serum, it is commonly used for determination and viability of early pregnancy [1]. The hCG structure is divided into two main subsets (α and β) with the former identical to that of LH, follicle stimulating hormone (FSH) and thyroid stimulating hormone (TSH) subsets, while the latter is specific to hCG.

HCG levels significantly increase during early pregnancy with levels doubling approximately every 48 hours [2]. This yardstick has become of importance in identifying cases of possible poor pregnancy outcome (for exemple, early miscarriage, ectopic pregnancy) [3-13]. However, the accuracy of using hCG to determine long-term pregnancy outcomes has been little investigated in women with a history of infertility and pregnancy following ovarian stimulation and exogenous luteal phase support. Therefore we wished to determine if early β-hCG determination can accurately forecast the outcome of ICSI pregnancies.

## Materials and Methods

### Patient population

This retrospective study was approved by our institutional review board. We performed a computerized search of our unit's patient record database for women who underwent ICSI at our center, had a positive initial β-hCG on day 14 post embryo transfer pregnancy test, and were followed up until the end of the pregnancy. Two hundred and sixty-one women met our inclusion criteria and were identified for analysis.

### ICSI protocol

Our center's ovarian stimulation and embryo transfer protocols have been published previously [14, 15]. In brief, women were down-regulated using a gonadotropin-releasing hormone agonist (Decapeptyl, Ferring NV, Belgium) protocol, followed by ovarian stimulation using recombinant FSH (rFSH, Puregon, NV Organon, Oss, The Netherlands) and/or human menopausal gonadotrophin (Menogon, Ferring NV, Belgium) till the day of hCG administration. When the leading follicle reached about 18 mm in diameter, 10,000 IU of hCG (Pergnyl, NV Organon, The Netherlands) was given intramuscularly, and oocyte retrieval was performed 34 - 36 hours later. Embryo transfer was performed on day 3 using a standardized technique using a soft Edward-Wallace or Cook catheter with the embryos deposited about 1 cm from the uterine fundus. Luteal phase support is provided in the form of daily progesterone vaginal suppositories every 12 hours (Cycologest 400 mg; Hoechst Roussel Limited, UK).

It is important to note that even the vast majority of patients in each group were less than 35 years old, an average of about 3 embryos were transferred since, as a referral center, most of our patients had previous failed trials in different centers.

### Hormone assays

Samples of venous blood were collected routinely two weeks post-ET. Serum β-hCG concentrations were measured by Mini Vidas, Biomereux, Italy. Patients who became pregnant following ICSI were followed up until the end of pregnancy at our center.

### Outcome measures

The primary outcome measure for this study was to determine the accuracy of early β-hCG in predicting the occurrence of a live-birth. Additionally, we wished to determine the accuracy of early β-hCG in predicting the occurrence of an ongoing pregnancy, a late miscarriage, an ectopic pregnancy, or an early miscarriage following ICSI. Live-birth was defined as the live-birth of an infant at 20 or more completed weeks of gestation. Ongoing pregnancy was defined as an intrauterine live fetus 14 weeks or more of gestation. Miscarriage was defined as pregnancy termination prior to 20 weeks gestation as confirmed by ultrasound, pregnancy test or by histology. Ectopic pregnancy was defined as an extra-uterine gestation as determined by ultrasound and confirmed by histopathology.

### Statistical analysis

All analyses of significance were two-sided and tested at the 5% level; values of P < 0.05 were considered to indicate significant differences. Continuous variables were tested if they presented normal distribution using the f-test. Accordingly, the Student t or Mann-Whitney U tests for parametric and nonparametric data, respectively, were applied. A receiver operating characteristic (ROC) curve was used to determine the β-hCG cut-off level that best discriminated between pregnancy outcomes. Clinical and demographic data are also presented as mean (SD) or as frequency distribution for simplicity. Statistical analysis was performed using the computer statistical package Stats Direct (Stats Direct, Ltd, UK).

## Results

The patient demographics and characteristics cycle did not differ between the groups with no significant difference with regards to patient age, period of infertility, and day-3 FSH levels (Table 1). Even so, the β-hCG levels were statistically significantly higher in pregnancies that reached the stage of an ongoing pregnancy and live-birth as compared to early pregnancy termination (Table 2). Additionally, pregnancies with an ectopic implantation or that had an early miscarriage showed significantly lower average β-hCG levels that pregnancies were viable for longer gestations. Pregnancies with late miscarriages had a lower average β-hCG level that pregnancies were viable for longer gestations, but this did not reach statistical significance.

**Table 1:** Patient Demographics and Cycle Characteristics Between Pregnancies That Reached Different Stages of Pregnancy

	Live-birth	Ongoing Pregnancy	Late Miscarriage	Ectopic Pregnancy	Early Miscarriage
Age	29.54 ± 5.05	29.61 ± 4.91	29.69 ± 5.29	30.4 ± 1.67	30.8 ± 4.83
Infertility duration	7.9 ± 3.97	8.34 ± 4.21	7.85 ± 4.43	10.4 ± 3.13	8.2 ± 5.23
No. of ampoules	51.59 ± 256.71	46.4 ± 230.91	27.87 ± 7.62	23 ± 7.42	29.71 ± 12.27
Days of stimulation	9.78 ± 1.86	9.78 ± 1.8	10.15 ± 2.92	9.2 ± 1.92	9.87 ± 1.73
No. of oocytes retrieved	12.48 ± 7.52	12.13 ± 7.33	13.25 ± 9.89	12 ± 6.44	11.8 ± 7.32
No. of MII	10.04 ± 6.47	9.76 ± 6.28	10.79 ± 8	10.6 ± 6.23	10.33 ± 6.77
Injected	10.12 ± 6.41	9.85 ± 6.22	10.71 ± 7.22	10.6 ± 6.23	10.33 ± 6.77
Fertilized	7.04 ± 4.51	6.78 ± 4.45	7.03 ± 5.48	5.4 ± 4.72	7.73 ± 5.57
Good embryo G1	4.15 ± 3.4	4.01 ± 3.23	3.94 ± 3.75	3.6 ± 2.88	5.2 ± 5.73
Fair embryo G2	2.43 ± 2.19	2.37 ± 2.07	2.49 ± 2.45	2.4 ± 2.61	2.33 ± 1.68
Poor embryo G3, G4	1.22 ± 1.76	1.14 ± 1.66	1.29 ± 1.67	0 ± 0	0.6 ± 1.35
No. of ET	3.75 ± 0.81	3.74 ± 0.83	3.76 ± 0.83	3.2 ± 1.3	3.4 ± 1.06

**Table 2:** Early Day 14 β-hCG Levels Between Pregnancies That Reached Different Stages of Pregnancy

	Live-birth*		Ongoing Pregnancy*		Late Miscarriage		Ectopic Pregnancy*		Early Miscarriage*	
	*Present*	*Absent*	*Present*	*Absent*	*Present*	*Absent*	*Present*	*Absent*	*Present*	*Absent*
Number of pregnancies	136	125	169	92	72	189	5	256	15	246
Mean SD	1070.02 ± 1505.79	686.6 ± 1269.69	1081.14 ± 1505.42	528.64 ± 1132.24	650.45 ± 1249.53	976.27 ± 1457.17	113.89 ± 65.42	901.48 ± 1417.7	82.21 ± 249.33	935.43 ± 1434.87
Significance	P = 0.0277		P < 0.0001		P = 0.0948		P = 0.0162		P < 0.0001	

*Statistically significant

**Figure 1.**
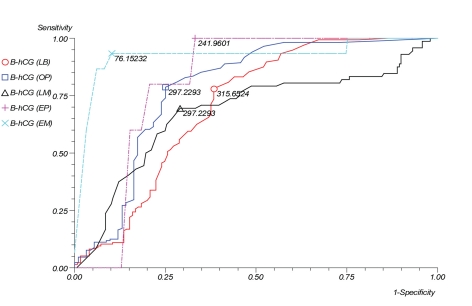
β-hCG cut-off levels on receiver operating characteristic (ROC) curve that best discriminated between pregnancy outcomes.

The results of the ROC curves for each stage of pregnancy termination demonstrated revealed a high sensitivity for identifying patients with ectopic pregnancies and early miscarriage (100% and 93.33% respectively) (Table 3, Fig. 1). The remaining results ranged from a sensitivity of 69% to 79% and specificity of 62% to 75%.

**Table 3:** β-hCG Cut-off Levels on Receiver Operating Characteristic (ROC) Curve That Best Discriminated Between Pregnancy Outcomes

	Cut-off	Area Under ROC	Sensitivity	Specificity
Live-birth	315.65	58.14%	77.94%	61.60%
Ongoing pregnancy	297.23	62.92%	78.70%	75.00%
Late miscarriage	297.23	67.72%	69.44%	70.90%
Ectopic pregnancy	241.96	81.17%	100.00%	66.80%
Early miscarriage	76.15	91.19%	93.33%	89.84%

## Discussion

The early weeks of fetal development are highly sensitive to changes in the embryonic milieu. Pregnancy survival past the initial critical weeks of embryonic development depends upon proper functioning of both the maternal and fetal endocrinological systems. Any imbalance may result in failure of the pregnancy to become viable and reach the stage of full embryonic development. Since β-hCG levels represent trophoblastic mass and function in the early fetal stages, its maternal serum concentration grows in a linear pattern to fetal growth and development with any divergence from this predefined incremental curve a suspicious sign of fetal developmental abnormality [16-18]. Low initial β-hCG values have additionally been linked to a poor prognosis [3-13]. Even so, what remains unclear is whether this highly accurate test for determination of early pregnancy demise is as accurate for long-term pregnancy outcomes.

As compared with the general population, early pregnancy monitoring is more highly emphasized in patients who become pregnant following assisted reproductive technologies (ART). Early pregnancy complications including ectopic pregnancies, abnormal fetal development, and chromosomal aberrations are higher in ART babies than naturally conceived children [19]. Additionally, couple's anxiety and the costs associated with ART make all children born using assisted reproduction 'precious babies'. These factors make the qualitative evaluation of ART pregnancies more important than their naturally conceived counterparts.

Measuring quantitative serum β-hCG has been used for predicting pregnancy outcome since the 1960s. Zegers-Hochschield et al [13] compared early hCG levels in patients who conceived naturally or through assisted reproduction and noted that the former had significantly higher hCG levels. Additionally, Confino et al [4] noted that the patients with poor outcomes (for example, miscarriage) had statistically lower hCG levels than normal pregnancies. Indirectly this may relate to a higher incidence of poor outcomes in ICSI pregnancies compared with naturally conceived pregnancies.

The predictive value of a single early β-hCG measurement on early pregnancy outcome has been studied by previous investigators [3-13]. Fridstrom et al [20] demonstrated that a day 14 post-ET β-hCG level of more than 150 IU/L had a 79% sensitivity and 78% specificity in distinguishing between viable and pathological pregnancies. Similarly, Bjercke et al [21] noted that pregnancies with a β-hCG level of more than 55 IU/L on day 12 had a 90% probability of reaching the stage of an ongoing pregnancy.

The results of this study demonstrate further support for the findings by previous investigators, and additionally, provide evidence of a correlation between early β-hCG levels and the probability of a positive pregnancy outcome. This may suggest that fetuses with higher initial production of β-hCG are more biologically competent and therefore more likely to reach the stage of delivery

In conclusion, in the current study we found that, in ICSI pregnancies, a single early β-hCG may help to identify pregnancies that will reach full-term and delivery. These results may help to reassure patients and help to identify high-risk pregnancies early on.
